# Malaria control by commodities without practical malariology

**DOI:** 10.1186/s12889-017-4454-x

**Published:** 2017-06-21

**Authors:** J. Kevin Baird

**Affiliations:** 1 Eijkman-Oxford Clinical Research Unit, Jalan Diponegoro No.69, Jakarta, 10430 Indonesia; 2 Centre for Tropical Medicine and Global Health, Nuffield Department of Medicine, University of Oxford, Oxford, UK

**Keywords:** Malaria, Prevention, Control, Strategy, History, Malariology, Species sanitation

## Abstract

Malaria remains a serious clinical and public health problem, the object of an ongoing technological and humanitarian struggle to abate the very substantial harm done. The manner by which humanity approached malaria control changed abruptly and profoundly after 1945 with the advent of the insecticide DDT. Malariologists in the first half of the twentieth century conceived precise modifications to natural or man-made environments aimed at making those less hospitable to specific anopheline mosquito vector species. This practical malariology achieved very significant reductions in burdens of morbidity and mortality, but the revolutionary insecticide eliminated the need for its specialized knowledge and diverse practices. By 1970 mosquito resistance to DDT and perceived environmental concerns precipitated the collapse of what had been a vigorous global campaign to eradicate malaria. Humanity did not then revitalize practical malariology but turned to another commodity as the foundation of control strategy, the war-spurred suite of synthetic antimalarial drugs developed in the 1940s and 1950s. When those drugs became lost to parasite resistance in the latter twentieth century, malaria resurged globally. Since 2005, tens of billions of dollars mobilized new commodities to control malaria: point-of-care diagnostics, effective artemisinin-based treatments, and longer-lasting insecticide treated bed nets. The know-how of practical malariology is not part of that ongoing commodities-based strategy. This article examines contemporary malaria control in the broad strokes of a strategy mitigating the consequences of infection contrasted to that of the abandoned practical malariology strategy of prevention. The inherent risks and limitations of over-reliance upon commodities in striving to control malaria may prompt consideration of a strategic posture inclusive of the proven methods of practical malariology.

## Background

Malaria imposes a serious threat to public health across large swathes of the globe where endemic transmission still occurs. Nearly 3 billion people live at risk, with hundreds of millions infected and hundreds of thousands not surviving the infection each year. The history of rational evidence-based efforts to control malaria over the past 120 years presents a patchwork of varied strategic thinking and outcomes colored by misplaced human reason, optimism, and impatience. We know much of the intimate details of the lives of these parasites and their mosquito hosts. Although we possess technology that safely kills them both, malaria continues to sap and kill us on those extraordinary scales. Asking why and how that occurs requires examining the tools, tactics and strategies brought to bear in striving to control malaria. Are those sensibly the best means available to us to combat this dangerous and complex problem?

Exploring that question necessarily considers the biological mechanics of malaria. This parasitic infection is only rarely contagious (e.g., congenital) and requires a mosquito to transmit it among humans. In a strict biological sense, humans are the intermediate hosts, a vehicle of transmission to the mosquito definitive host where sexual reproduction occurs. The British Indian Army surgeon, Ronald Ross, made that discovery in 1898 and created a rational basis for structuring public health campaigns against a malaria problem – attack the mosquito carriers and *prevent* the infection rather than simply treat the infected and ill. Malariology in public health is that science and method.

This article posits malariology as discarded from the proverbial toolbox of malaria control. Many workers today engaged in the struggle against malaria may view their expertise as malariology, just not as Ross had conceived it. It may be reasonably argued that malariology evolved out of its original character rather than vanished. Putting those semantics aside, the loss of that character is what matters here. Accepting the term practical malariology for that as Ross conceived it serves as a useful conceptual device separating the ideas, tools, and actions that prevent rather than mitigate harm done. This important distinction provides greater clarity of technical and strategic postures in the control of malaria.

The loss of practical malariology may be appreciated by assessing the human defenses now in place against endemic malaria. These represent the sum of our humanitarian spirit, collective knowledge, technological ingenuity, and strategic thinking – all we have to pit against an often-mortal threat occurring in the environment. At almost any malarious site at the most remote locations, one encounters modern rapid diagnostic devices, effective drugs, and insecticide-treated netting. Historic generosity mobilized these over the past decade. But inventing and using these objects to mitigate harm is not practical malariology. The know-how of preventing harm by rendering the environment less hospitable to anopheline vectors will not be found at malarious sites today because we abandoned those methods 70 years ago.

Public health commits to a core principle of preventing disease. Practical malariology strived to achieve that by specific alterations to the environment disfavoring anophelines. These actions aimed to disable the environment as an agent of disease by attacking the capacity to support ready carriage of malaria. Contemporary malaria control mitigates harm but without that offensive weapon of practical malariology. This strategic posture traps us in a perpetually defensive position against the endemic malaria foe. We crouch behind insecticidal parapets diagnosing and treating the infected, hoping those defenses outlast the anopheline-plasmodial assault upon us. Sometimes it does, but far more often it does not and our material defenses erode by irresistible Darwinian processes. This article critically examines the history, technology, and strategic thinking underpinning malaria control without practical malariology and advocates resumption of its practices in order to enable an offensive strategic posture against endemic malaria.

## Practical malariology

“*I have said elsewhere that the Panama Canal is being dug with a microscope, and I believe the same instrument will double the wealth of the Federated Malay States*.”

Ronald Ross, 1911.

The quote prefaces Watson’s monograph on malaria control in the British Federated Malay States (modern Malaysia and Singapore) [1]. Ross expresses the power of practical malariology, enabled just 13 years prior by his discovery of the mosquito-borne transmission of malaria. In this era Ross vigorously battled powerful advocates of mass quinine treatment for malaria control (chiefly the preeminent Robert Koch). Watson’s spectacular successes with vector-based malaria control countered the complete failure of similar efforts by Ross at Mian Mir in the Punjab of modern Pakistan [2].

Decades of broader successes followed in Panama, Brazil, India, China, British Malaya, the Netherlands East Indies (modern Indonesia), the African continent, and in southern Europe and the United States. This practical malariology required careful scientific study of mosquito bionomics in malaria transmission, engaging the expertise of specialists called malariologists plying interventions necessarily tailored to singular local transmission ecologies. In 2005 Keiser and colleagues [3] conducted a systematic analysis of 16 malaria control interventions (most conducted before 1945) involving environmental modifications. They found the burdens of malaria reduced on average by 88% (95%CI 82–92). This malaria control strategy rooted in the practical malariology of Ross had been vetted, honed, and vindicated as an effective means of preventing and sometimes eliminating malaria by 1940. The specific character of some of these interventions will be described later in this article.

Discovery and application of the seemingly miraculous insecticide DDT on enormous scales by the late 1940s [4] rendered practical malariology redundant. Mechanical DDT usage ushered in the age of an industrial commodity-driven malaria control applied in the same fashion almost anywhere. Malariology as Ross and others conceived and developed it – bespoke interventions derived from ecological studies of great exactitude – came to be seen as unnecessarily laborious and irrelevant to the control of malaria by the 1950s. Although the technology of practical malariology had not yet peaked in impacts, World War II broadly arrested its practices and the subsequent rise of DDT averted resumption of its further development. Today, the practical malariologists capable of plying their entomological know-how in attacking entrenched malaria have very nearly vanished.

Allowing the decline of practical malariology may have been a historic strategic error. If so, we seem unable to recognize and reverse it. We abandoned complicated practical malariology and embraced the relative ease and simplicity of generic malaria control by DDT and antimalarial drug commodities. We continue living with that strategic shift, but with newer insecticides and drugs. Comparing and contrasting the tactics of contemporary malaria control with and without practical malariology more fully illuminates the loss and its strategic implications.

## Malaria control strategy

Human malaria is not a single infection but a number of them acquired from any of several dozen mosquito vector species in the genus *Anopheles* transmitting at least five protozoan species in the genus *Plasmodium* [5]. Collectively, these malarias represent a very complex problem of global health requiring actions that mitigate harm and attack its sources. Those varied actions compose what is called malaria control, and distinct strategies in turn define tactical actions formulated upon contemporary thinking and available technology. Rational malaria control has seen three major strategic focuses: environmental (1900–1940), insecticidal (1950–1970), and therapeutic (1980-present). The shift in each saw a Nobel Prize awarded to the enabling discoverer: Ross (mosquito transmission, 1903), Paul Müeller (DDT, 1949), and Youyou Tu (artemisinin, 2015). We rightfully attach great significance to our abilities against the malaria problem, and, for better or worse, the award lends great weight of validity to the technological approach in striving to solve it.

Diagnosis and treatment have dominated as the strategic core of contemporary malaria control over the past 40 years. Commodities called rapid diagnostic tests (RDT) and artemisinin combined therapies (ACT) serve as the current physical elements of the tactical actions of the strategy. The addition of longer lasting insecticide-treated nets (LLIN) and revitalized indoor residual spraying (IRS) of insecticides to that toolbox since around the new millennium adds a modest dimension of prevention [6]. Some may think of LLINs or IRS as a vector control strategy, but it is one limited to killing mosquitoes seeking out humans sleeping indoors. These do not impact early or outdoor feeding/resting mosquitoes, nor, most importantly, do they impair the ability of the environment to support an anopheline population of any behavioral phenotype.

Practical malariology leveraged understanding of transmission ecology in formulating interventions aimed at minimizing the capacity of the environment to stabilize local anopheline populations in numbers sufficient to sustain transmission [7]. In the age of Ross, the malariologist on the scene made real time assessments regarding tactics suited to the site under consideration – discovering and exploiting anopheline vulnerabilities in the environment. In the current age, there is almost never a malariologist on the scene. The people at those sites have training in using RDT, ACT, and LLIN/IRS, but rarely any more than fundamental knowledge of malaria as a problem rooted in highly variable but very specific features of the environment. Those commodities are distributed to where needed with the hope of mitigating harm, but without understanding the specific source of it in the environment. In plying the tactics of contemporary malaria control strategy, ecological vulnerabilities in transmission thus go unnoticed or unexamined and ultimately unexploited. Anopheline species carrying malaria thus often enjoy a virtually undisturbed habitat and fecundity in endemic communities.

Viewing the community as the patient of public health, contemporary malaria control addresses the symptoms of being malarious – infected people and infectious mosquitoes. The source is a stable population of vector mosquito species sustained by specific features of their environment in that community. Public health interventions against infectious threats are not limited to treating the ill or minimizing exposures, but include disabling its source as an agent of disease. Contemporary malaria control surrendered the latter and relies almost solely upon the former.

The examination of scientific versus practical malariology to follow explores the question of how and why malaria control strategy today effectively disregards the environment as an opportune target. The strategic shift to DDT and new synthetic antimalarial drugs occurred in an age of revolutionary scientific advancement, and these commodities then failed at the dawn of the biotechnological revolution. These historic contexts matter. Great faith and hope in scientific innovation colored strategic outlooks for combatting malaria. Laboratories shone as the founts of solutions to heretofore-unsolved medical problems. The front line of our struggle against malaria – where humanity places its most precious capital of will and intellect – thus became and remains the work of those laboratories. We look to them in the crucially important task of creating the commodities applied in controlling malaria. Although science has indeed delivered these, this posture nonetheless carries important strategic implications in malaria control, and perhaps steep risks and intrinsic limitations.

## Scientific malariology

Design of the objects used in malaria control represents the only opportunity to exploit recognized parasite/mosquito vulnerabilities within that limited strategic scope. A large and robustly funded community of scientists strives to bring better commodities to that enterprise – principally vaccines, diagnostics, drugs, and insecticides. Their work encompasses varied arrays of technological endeavors, many at the frontiers of science. Most labor far from active malaria transmission in superbly outfitted laboratories. This very important cutting-edge work is not practical malariology but may be broadly characterized as scientific malariology.

Whereas practical malariology engages the problem of malaria in real time in the real world employing proven strategies and tools, scientific malariology explores unknown and unproven technologies aimed at later improving the practice of malaria control. Scientific malariologists develop the physical tools of malaria control, very often by conceiving an object – be it a molecule or device – intended for global application. Those scientists thus share a broad strategic view of malaria control as a commodity-driven industrial enterprise generating objects impacting parasites or mosquitoes no matter where they occur or how distributed in highly diverse habitats. The tools are thus necessarily generic, directly targeting the organisms without regard to hyper-variable vulnerabilities in their environmental placement or physical needs in malarious communities. The commodities of scientific malariology therefore have little utility to the primary method of practical malariology – specialized knowledge of local anopheline bionomics shrewdly exploited against them. Scientific malariology thus conceives and optimizes generic objects of an intrinsically defensive character due to an imposed overarching strategic limitation: an inherent aim at the problem rather than its source.

Complex and varied reasons explain our very strong affinity for generic commodities over environmental actions for malaria control. Apart from the relative ease and simplicity of production, distribution and application, these represent perhaps the most direct means available to the scientific/industrial communities of North America and Europe for engaging a distant malaria problem. They are less inclined to consider practical malariology as a means of engagement – ideas, know-how, and practices being less exportable than objects. Moreover, competence and mastery of the discipline can only be cultivated by direct and prolonged exposure to the problem as it occurs naturally.

That know-how is not being cultivated in endemic areas on any scale near to adequacy to the problem. The conceptual, practical, and subsidized economic ease of malaria control by commodities colors the strategic outlooks of producers and users alike. Each adopts the view of malaria control as the relatively simple enterprise of distributing useful objects to where and when needed. Opportunity for altering the environment against anophelines thus loses traction or appeal as a relatively demanding and complicated strategic option. Recognizing this as a problem to be addressed first requires acknowledging the intrinsic risks and strategic limitations of being solely reliant upon objects for malaria control.

## Pitfalls of malaria control by commodities

There is no doubt that the four principal commodities of contemporary malaria control – RDT, ACT, IRS, LLIN – greatly dampen morbidity and mortality. Likewise, we may be certain these do not attack the source of the problem beyond killing mosquitoes encountering insecticides where people sleep. Moreover, the varied ecologies and human behaviors impacting malaria transmission may impair the impacts of these commodities. Each is sometimes a poor fit or under threat of loss, as capsulized below.

RDT -- Chronic exposure to malaria generates asymptomatic carriers of sub-patent parasitemias beyond the diagnostic reach of RDTs. The lower the prevalence of patent infection, the higher the proportion of sub-patent infections [8]. As nations make headway in malaria control, RDT diagnostic efficacy wanes. This tool fails to detect most asymptomatic infections in low intensity ecologies of transmission, though it reliably diagnoses acute malaria. Most RDT brands rely on antibody capture of parasite antigens known as histidine-rich proteins 2 and 3, but mutants lacking these proteins occur in the wild [9, 10]. Natural selection of them, i.e., by slowed or failed diagnosis and treatment may be anticipated where RDT coverage is relatively high. These diagnostics are under threat of loss.

ACT – These drugs do not impact the hypnozoites of *Plasmodium vivax*. The latent hypnozoite reservoir of that infection requires a drug called primaquine. That treatment is often not applied due to dangerous toxicity to patients having an inborn deficiency of glucose-6-phosphate dehydrogenase. That deficiency occurs at a prevalence of about 8% in malaria endemic nations [11]. Over 80% of acute attacks of *P. vivax* in endemic areas derive from hypnozoites [12, 13]. ACT therapy alone thus will not suffice to control or eliminate malaria transmission [14]. Moreover, evidence of parasite resistance to ACT now occurs across much of the Greater Mekong Sub-Region [15]. These drugs are under significant threat of loss and newer drugs are not yet available.

IRS/LLIN -- The anopheline vectors exhibit a wide range of generally species- specific feeding behaviors [16]: some strongly prefer humans, others may prefer domesticated animals or opportunistically feed on most abundant hosts; some typically feed indoors, whereas others rarely do; and peak feeding hours may occur in early evening before people retire or in the early morning after they become active. If an anopheline species naturally bites outdoors or in the early evening/morning, it may be unlikely to encounter LLIN or IRS [17]. Anophelines in many regions show either resistance to the insecticides of LLIN and IRS [18], or have adopted modified feeding response behaviors shifted away from exposure to those objects [19]. These primary means of reducing human contact with anopheline mosquitoes are often inadequate to local anophelines and under threat of loss to physiological or behavioral resistance.

This suite of commodities is not universally effective or exempt from loss either by biological, logistical, or economic failures. The pursuit of improved commodities to replace them remains critically important and today engages the energies and resources of the vast majority of the community of scientific malariology. The risk is a gap in time between failure and replacement, opening a door where malaria may catastrophically rebound as it did in the late twentieth century. When commodities thus fail or vanish, how to then deal with an unsolved malaria problem?

### Malaria control without commodities

Malariology advanced tremendously through the first half of the twentieth century, developing effective interventions against endemic malaria by making the environment less hospitable to anopheline vectors. Where applied and reported, these interventions reduced malaria burdens by an average of 88% without modern commodities [3]. These outcomes required only local expertise to conceive, optimize and implement in order to realize prevention impacts. That expertise being exceedingly rare today largely explains its absence from contemporary malaria control practices. Explaining our apparent satisfaction in working without practical malariology is a more difficult proposition.

The complex ecologies of malaria transmission are as varied as the mosquito, parasite, and human players involved, but very specific environmental features ultimately define where, when, and in what numbers anophelines occur. Most anopheline species thrive under a rather narrow range of conditions favored by it [20]: some bloom with onset of rainy season, others do so during dry season; some require shade, others require direct sunlight; some tolerate brackish water while others do not; and many require very specific and relatively pristine breeding sites, while others utilize almost any temporary depression like a rain-filled tire rut or hoof print in a muddy pasture. The strict physical requirements of the aquatic larval stages of anophelines often represent profound vulnerability to destruction.

Attacking larval anophelines requires very specific knowledge of taxonomic species identity (*define* appropriate target), importance in malaria transmission (*incriminate* target of impact), where and when it occurs (*locate* for physical targeting) and finally conceiving a means of prosecuting an attack (*implement* measures). Attack may consist of modifying the environment to physically rid it of breeding sites. More permanent sites may be rendered inhospitable by flushing or clearing, or placing biological agents (pathogenic microbes, or predatory insects or fishes) or chemical larvicidal agents at those sites. These varied modes of attack all aim at reducing the ability of the environment to support a stable population of incriminated local vector anophelines.

At British Malaya in the 1900s Malcolm Watson invented the control of malaria by implementing specific and often subtle environmental modifications aimed against a particular vector species of anopheline [2]. This approach became known as “species sanitation”, i.e., sanitizing the environment against a particular species of mosquito. Watson halted malaria transmission by shade-loving *Anopheles umbrosus* simply by removing shade trees [21]. During the years 1919 to 1932 at a site in West Java, spleen indices (proportion of residents having hemozoin-laden enlarged spleens due to chronic exposure to malaria) uniformly exceeding 85% were reduced to <5% simply by periodic and repeated flushing of rice paddies when maximal larval densities of *Anopheles aconitus* occurred in them [22]. Similar results were obtained by removing surface algae mats protecting *Anopheles sundaicus* larvae from predation in commercial fishponds all across coastal Java [22]. More stubborn hillside malaria carried by *Anopheles maculatus* yielded to precisely- timed flushing of rocky streams [23]. Many other successful species sanitation operations occurred [3], including against the notoriously difficult African vector, *Anopheles gambiae* [24, 25].

In 1913 Watson visited Nicholas Swellengrebel in neighboring Netherlands East Indies Sumatra, alerting him to species sanitation methods [2]. Swellengrebel and his Indonesian protégé’ Raden Soesilo (Ph.D. student of W.A.P. Schüffner) went on to perfect species sanitation practice for malaria control [22]. In so doing, they brought anopheline taxonomy into the realm of public health [26]. Swellengrebel and DeBuck [27] expressed in 1938, “*In the Far East matters look hopeful. Out there,* Anopheles *belong to many species, some of them dangerous and many others harmless. Each one of them has its own peculiar habits, and so we can pick out and destroy the dangerous species, while leaving the harmless ones unscathed. Without practicing species sanitation one feels hopeless… If anophelines are to be controlled by species sanitation, they should belong to a taxonomic unit which remains constant in all circumstances. It is useless to apply species sanitation to the control of an inconstant taxonomic unit…”.*

Anopheline taxonomy today seems an esoteric endeavor divorced from malaria control and health dividends. In practical malariology, taxonomic precision guides the primary offensive actions against endemic malaria. Modern molecular genetic studies of anophelines unveiled species complexes, seemingly morphologically identical mosquitoes being composed of closely related ‘sibling species’. Distinct members of the same complex sometime exhibit unique bionomics relevant to malaria transmission. This introduces a layer of complexity to the task of defining targets, but also offers opportunity for greater precision in striking at them. At present, however, the principles and practice of species sanitation remains alien to most workers in malaria science and control. Attacks of any precision almost never occur.

Some species sanitation interventions still prevent malaria nearly a century later. *Anopheles aconitus* no longer threatens rice cultivation areas of Java because the strategy implemented – flushing paddies at opportune times – became permanent agricultural practice. That species is a relatively inefficient carrier requiring great numbers to sustain malaria transmission. The implementing malariologists thus did not impractically aim to eradicate *An. aconitus*, but instead to drive numbers down to non-threatening levels. That species still occurs on Java but in benign numbers. There may be species of anophelines in some settings not amenable to species sanitation due to the manner or scale of environmental modifications required [25]. Today the competency needed to make local assessment of practicality is simply too rare for wide practice.

We can examine the older literature from the early malariologists reporting extraordinary successes on massive scales. Although there may well be a reporting bias favoring successes over failures in that literature [3], the evidence minimally offers proof-of-concept that species sanitation or other environmental approaches achieved great impacts in varied habitats across relatively wide geographic areas. Knowledge of this evidence begs a crucial question of contemporary malariology: at high efficacy, simplicity of implementation, sometime permanence of impact, and without costly or complex technologies, what obstacle discourages resumption of these practices? It cannot be impracticality – they were practiced. It cannot be ineffectiveness – they were effective. It cannot be awaiting enabling technological advances – the technology is in hand.

The obstacle may be the overwhelmingly dominant imperatives of scientific malariology and the ever-present hope of incipient technological advances enabling greater progress with relative ease. Consider how we might receive a vaccine of 88% sterilizing protective efficacy and contrast that with the utterly abandoned environmental methods demonstrably capable of achieving the same impact. The logic and rationale at work in discarded practical malariology may be very hard to discern and grasp, but its consequences are not.

## Strategic shifts

The advent of DDT decimated practical malariology and species sanitation in malaria control. Humanity had opted for the simplicity of a single approach to malaria control grandly aimed at global eradication. This failed insecticidal enterprise formally collapsed by 1970 [28, 29], and a broad public and scientific apathy for the malaria problem followed. We surrendered aspiration for eradication and weakly resumed conventional malaria control without practical malariology. Broad disillusionment with vector control with failure of the insecticidal strategy caused most control programs to neglect or abandon entomologic expertise [30].

After 1970 we turned to the sole remaining commodity, the chemotherapeutics composed principally of the suite of synthetic antimalarial drugs developed just before, during, and immediately after World War II. Malaria control became reduced to mitigating harm by diagnosis and treatment of the infected rather than any substantive prevention [31]. Research on new antimalarials had slowed to very few small laboratories, mostly those of military China and America. Chemotherapeutics development for malaria had largely vanished from mainstream biomedical research by the 1980s despite mounting evidence of the failure of available drugs to widening parasite resistance to them.

The same era saw the great strides of the biotechnological revolution spawning the immediate and captivating promise of an effective vaccine against malaria [32]. An end to the grindingly hard work of conventional malaria treatment and control perhaps seemed at hand, and basic biomedical research on malaria in the latter twentieth century greatly expanded in step with the broader revolution of that technology. Important advances indeed were made in understanding the biology of these parasites, much of it narrowly focused on that promise of a useful vaccine. 50 years later that product has yet to materialize, but by the new millennium research strategy had shifted priority back to urgently needed diagnostics and therapies as the global resurgence gained alarmingly [33, 34].

As vector control capacities withered in the latter twentieth century, so too did the efficacy of the few available drugs against malaria. Without vaccines or effective drugs, and an almost complete inability to combat anopheline vectors, malaria resurged powerfully throughout that era. We were not as adaptive as the parasites and countless lives perished in that onslaught. We cannot know if a revived and vigorous practical malariology would have averted this catastrophe, but that history nonetheless instructs on the peril of malaria control by commodities without alternative real world strategies to then cope with failed technological aspirations.

The late research efforts and extraordinary generosity afforded humanity a response to resurgent malaria. Enormous quantities of RDT, ACT, and LLIN have been mobilized since 2005. Morbidity and mortality have been significantly reduced, by as much as 37% and 60%, respectively [35], but these essential and conspicuously important tools are expensive (about $2.5 billion annually). These commodities are also intrinsically defensive, sometime ineffective, and vulnerable to biological loss or economic setbacks. Over-reliance on these objects, or placing hope in the appearance of newer and better ones, amounts to a gamble of extraordinary stakes. And it is one we had clearly lost just two decades ago.

Our commodities today compose a tenuous defense inadequate to a necessarily offensive strategy against this stubborn and deeply entrenched problem. Failed aspirations, over-reliance on objects, and the abandonment of practical malariology nakedly exposed humanity to catastrophic consequences. In a strategic sense, little has changed and we remain vulnerable to the same wretched outcome by the same causes [36]. The predictable, inevitable, and irreversible loss of a commodity may not be the core strategic problem – it may be the irrational, avoidable, and reversible loss of practical malariology to then be able to prevent harm with failed aspirations.

## Real world malaria

Aspiration for the eradication of malaria rightfully embeds in our awareness and strategic thinking on the problem. We cannot accept mitigation alone as a satisfactory solution, though our current toolbox weakly offers only that. Science may be very likely to deliver powerful new tools, but when it may do so is the operative question. Human nature places that bright horizon near to an ever-shifting present, as it has been since the discovery and application of DDT a human lifetime ago. Such hope may be characterized as reckless hubris without acknowledging the possibility of failed aspirations and a cultivated means for then effectively managing an unsolved problem.

Rational pragmatism demands strategic plans for coping with malaria well into the long balance of the current century. Many of us witnessed firsthand the awful consequences of failed aspirations during the 1990s and early 2000s, material and strategic defenselessness against rampant malaria morbidity and mortality. Onerous real world malaria – stubborn and adaptive – commands the respect needed to exceed those qualities in combatting it. Impatient hope weakens that resolve, as the history of our engagement with this foe instructs. The appeal here to deliberate cultivation of practical malariology in this battle grimly accepts the possibility of failed near-horizon aspirations. It looks to a malarious future in need of the lost malariology of Ross. The quintessential practical malariologist Leonard Jan Bruce-Chwatt [9] warned nearly four decades ago in the early years of resurging malaria, “*It is a fact that the older generation of malariologists has largely disappeared and the number of professional workers in tropical community health competent in the field of malaria control is woefully inadequate, while the need for them is steadily growing*.” That unheeded warning remains as relevant today.

No public or private institution offers training or certification of competency in practical malariology specifically, or even malaria control generally. Training in malariology occurs incidentally within mainstream medical and scientific research and practice dominated by a biomedical technological agenda. This largely explains the great abundance of scientific malariologists relative to the nearly extinct practical malariologists within the community of scientists engaging the malaria problem. We seem strategically oriented away from the real world malaria Bruce-Chwatt implored us to address with practical scientific know-how in the affected communities. This problem may be solved by committing to the task of expanding that base of established knowledge.

## A single division

The metaphor of warfare in our struggle with malaria embeds in that history. Not long after the collapse of the global malaria eradication campaign, medical historian Gordon Harrison [37] reflected, “*Failure so universal, so apparently ineluctable, must be trying to tell us something. The lesson could be of course that we have proved incompetent warriors. It could also be that we have misconstrued the problem.*” The ultimate outcome of the then incipient global resurgence in malaria surely demonstrated both lessons. We need only absorb them.

In military parlance a division is a force of about 15,000 troops. Many armies field dozens of these, each deliberately and specifically trained and equipped for a particular type of warfare environment. Faced with a hostile foe in that environment, each soldier plays his or her role plying training and equipment against an enemy. The force moves and acts in accordance with a defined strategy capitalizing its unique skills and gear – placing those where and when they would be most useful realizes their purpose and utility. Nations invest enormous financial and human capital in these endeavors largely because real or perceived enemies threaten their security and welfare. Malaria may be viewed as such an enemy.

Let us imagine a single division of Bruce-Chwatt’s tropical public health and medical professionals empowered by governments having deliberate and specific training in practical malariology. People working at the front lines of battle all across the malarious globe, plying their know-how in direct engagement with the source of the problem. As a soldier carries many tools and abilities for most contingencies on a shifting battlefield, so too does the malariologist. As the primary offensive tool of the soldier is a rifle, entomological know-how is the primary weapon of the practical malariologist. The enemy is the mosquito species transmitting malaria and the mission is to seek out and destroy it and that which offers it succor. All else – diagnostics, treatments, residential insecticides – is secondary first aid for defensively coping with the failure of that primary mission.

This simple clarity of purpose and defined abilities in the right place at the right time epitomizes competency in the warfare against malaria. This strategy requires no knowledge or technology that we do not already possess. It requires only the will to execute it enabled by the courage and reason to accept that we may have indeed misconstrued a problem of public health as one of medicine. The solution to malaria in the many areas where it occurs today is certainly not palliative and labile commodities. It is perhaps not a miracle vaccine, drug, or insecticide awaiting discovery. It may be simple know-how and a mindset of aggressive attack imbued in people positioned to effectively apply those attributes against a mortal enemy. Armies do not win wars with first aid kits, as necessary as those may be, but with weapons of offense in the hands of appropriately motivated people trained and positioned to use them competently. Practical malariology as Ross conceived and developed it is that weapon of essential public health. We need to pick it up, climb over the parapet and see what we can do with it.

## Strategy

Scientific malariology carries its essential visionary endeavors forward. It may do so with acknowledgement of the improved commodities it seeks as aspirational and, once realized, intrinsically defensive and prone to loss or unavailability. A vaccine offering long-term sterilizing immunity would revolutionize control of malaria, but perceiving ourselves as defenseless without it is neither true nor helpful. That perspective stems from a narrow strategic view constrained by the misperception of commodities as our only strategic option. The only strategic option in play is not necessarily the only one. Practical malariology not being in play does not infer that it cannot or should not be applied. The evidence and argument presented here asserts the distinct advantages of practical malariology, but also acknowledges the obvious necessity of our commodities in malaria control. The strategic options are not between commodities and practical malariology, but between commodities with or without practical malariology.

The advocacy expressed here calls for reorienting a defensive strategic posture almost wholly reliant on commodities to one also reliant on the know-how of attacking malaria carriage by evidence-informed environmental interventions against important anophelines. The crippling shortage of that know-how is the primary challenge with such a strategic shift. Meeting that challenge may seem onerous from the perspective of object-oriented strategists and implementers steeped in the operational simplicity of their approach, but it is objectively simple – disseminate genuine know-how in practical malariology as broadly and deeply as possible where malaria lives. The failure to directly engage malaria in this manner is not a problem of abilities but of an immovable failed strategic posture.

Placement of the front line of our engagement with malaria within the laboratories of scientific malariology, as it has been for over 70 years, has been a strategic failure. Over-reliance on the intrinsically defensive and often aspirational commodities of that front failed us catastrophically, and seriously threatens doing so again. We struggle to mitigate the harm done by highly responsive and adaptable parasites and mosquitoes, believing and hoping our commodities can keep up whilst developing no adaptive strategy against failure of that aspiration. We should accept a history that demonstrates the commodities of scientific malariology alone being inadequate to solving the malaria problem and adapt to that lesson. Attacking endemic malaria requires organic engagement against it with a tool that we know works – a science-based practical malariology built upon the core public health principle of prevention. Such deliberate action against the largely unmolested sources of endemic malaria transmission in the many environments where it now occurs promises very significant gains. These may be won by the conceptually simple action of training and competency in the technology of practical malariology proven a century ago.

## Conclusions

Contemporary malaria control is an industrial technological enterprise focused on the creation and application of generic objects of global utility. Four of those effectively compose the toolbox of contemporary malaria control: RDT, ACT, LLIN, and IRS. Loss of any of these before science produces superior alternatives could precipitate another global resurgence in malaria morbidity and mortality. And it would be for largely the same reasons – over-reliance on commodities, real and envisioned, enabling the nearly complete neglect of practical malariology. Malaria control solely by technological commodities imposes great limitations and risks. In a public health sense, it is palliative rather than curative care for the patient, the malarious community. Empowering the people now battling malaria with practical knowledge of malaria control by evidence-informed actions in malarious communities protects from rebound when commodities fail or vanish, as occurred in Venezuela [38] – and it directly enables the necessarily local endgame of malaria elimination, as occurred in Sri Lanka [39]. Revitalizing malaria control by know-how in practical malariology where malaria occurs offers a means of greatly improving impacts while simultaneously mitigating the steep risks of failed commodities.

## Abbreviations

ACT: artemisinin combined treatment for malaria
DDT: dichlorodiphenyltrichloroethane, and insecticide
IRS: indoor residual spraying of insecticide for protection against mosquitoes
LLIN: long lasting insecticide-impregnated net for protection against mosquitoes
RDT: rapid diagnostic test for malaria
